# Disseminated Bacillus Calmette-Guerin infection following concurrent intravesical BCG therapy and immune checkpoint inhibitior therapy: a case report

**DOI:** 10.1186/s12879-025-11511-3

**Published:** 2025-11-13

**Authors:** Martin Plymoth, Charbel Wehbe C., Ross Harvey, Taryn Crighton, Indy Sandaradura

**Affiliations:** 1 Centre for Infectious Diseases and Microbiology, Western Sydney Local Health District, Sydney, NSW Australia; 2 Department of Clinical Microbiology, Umeå university, Universitetstorget 4, Umeå, 901 87 Sweden; 3 NSW Mycobacterium Reference Laboratory, Centre for Infectious Diseases and Microbiology Laboratory Services, Westmead hospital, Sydney, NSW Australia

**Keywords:** Disseminated BCG, Bacillus Calmette-Guerin, Immune checkpoint inhibitor therapy

## Abstract

Disseminated Bacillus Calmette-Guérin (BCG) infection is a rare complication of intravesical BCG therapy, affecting both immunocompetent and immunocompromised patients. We present a case of disseminated BCG infection in an 86-year-old man undergoing immune checkpoint inhibitor therapy for relapsed Hodgkin lymphoma and concomitant intravesical BCG therapy for non-muscle-invasive bladder cancer. The patient developed a progressive mycotic abdominal aortic aneurysm, psoas abscess, and vertebral osteomyelitis. *Mycobacterium bovis* BCG was confirmed via Polymerase Chain Reaction (PCR) and mycobacterial culture from a psoas abscess biopsy. Despite appropriate antimycobacterial therapy and multiple attempts at abscess drainage, his condition deteriorated, ultimately leading to his death. This case highlights the challenges of diagnosing disseminated BCG infection, which can mimic malignancy, and suggests a possible link between immune checkpoint inhibitors and BCG dissemination. Clinicians should maintain a high index of suspicion for BCG-related complications, particularly among those receiving immune-modulating therapies.

**Supplementary Information:**

The online version contains supplementary material available at 10.1186/s12879-025-11511-3.

## Introduction

Disseminated Bacillus Calmette-Guerin (BCG) infection is a rare but recognised complication of BCG administration used for vaccination against Tuberculosis [1], as well as for intravesical therapy in the treatment of early-stage bladder cancer [2]. It has been described in patients with primary immunodeficiencies [3], and acquired immunodeficiencies such as HIV/AIDS, haematological disorders, transplant recipients, and other immunosuppressive therapies [4–6]. Here we present a case of disseminated BCG after intravesical therapy for bladder cancer, with disease progression while concurrently undergoing immune checkpoint inhibitor therapy for a haematological malignancy.

## Case

A 86-year-old man presented to the emergency department (ED) with a 4-week history of progressively worsening lower back pain. His medical history included relapsed/refractory classic Hodgkin lymphoma (cHL), high-grade non-muscle-invasive bladder cancer, low-grade prostate cancer (Gleason 6; untreated), type 2 diabetes mellitus (without complications) and essential hypertension. He reported a non-severe allergy to penicillins. The patient was retired, having previously owned a business, and currently resided in a retirement village, where he was independent with activities of daily living. He was a non-smoker and consumed less than 10 grams of alcohol per week.

### Background

**Fig. 1:**
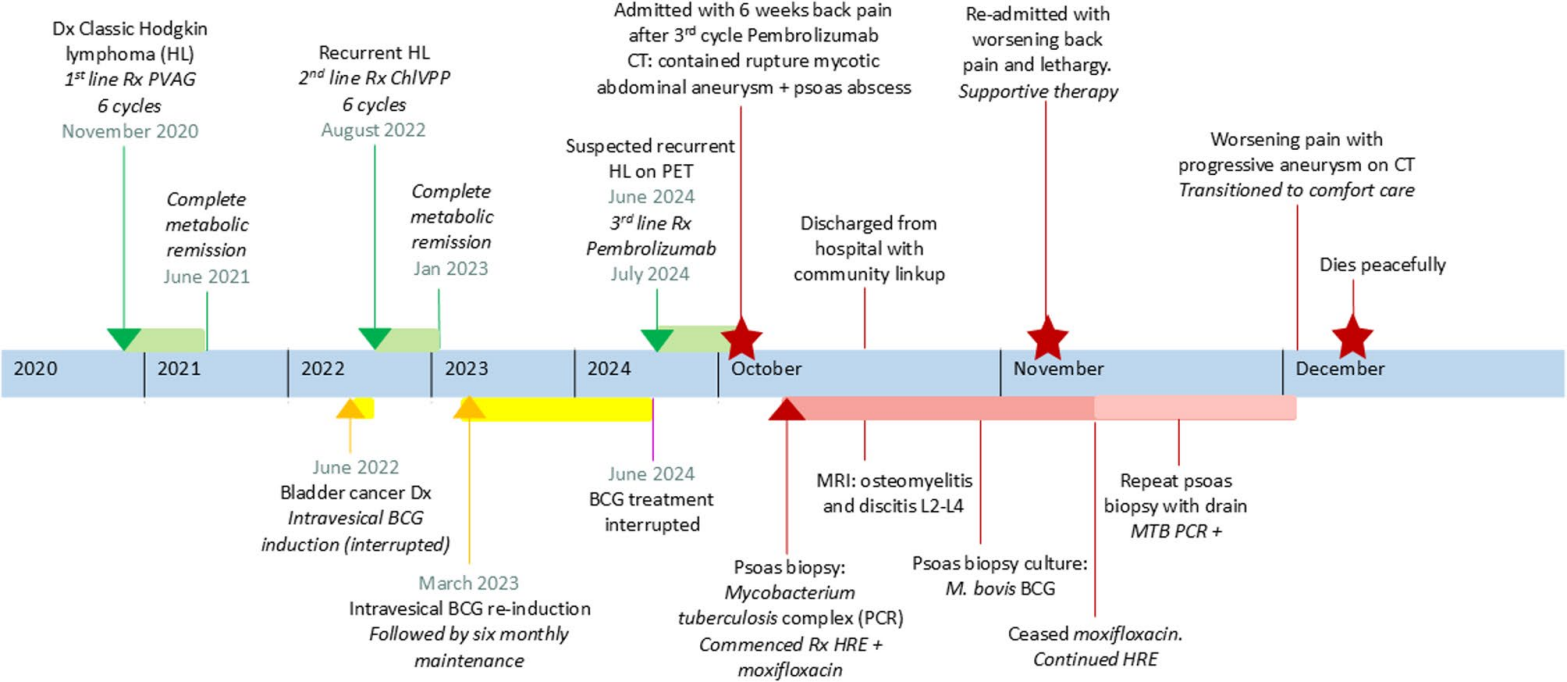
Timeline of events. Dx: diagnosis; Rx: treatment; HL: Hodgkin lymphoma; PVAG: prednisolone, vinblastine, doxorubicin, gemcitabine; ChlVPP: chlorambucil, vinblastine, procarbazine, prednisolone; BCG: Bacillus Calmette-Guérin; PET: Positron Emission Tomography; CT: Computer tomography; PCR: Polymerase chain reaction; MRI: magnetic resonance imaging; HRE: isoniazid, rifampicin, ethambutol; MTB: Mycobacterium tuberculosis complex

In 2020, he was diagnosed with biopsy-confirmed stage IV cHL, with extensive disease including paraaortic and hilar lymph node involvement, as well as liver lesions (timeline: Fig. 1). He underwent chemotherapy with prednisolone, vinblastine, doxorubicin, and gemcitabine (PVAG), achieving complete metabolic remission after six cycles.

In 2022, during a routine cystoscopy as part of prostate cancer workup, urothelial carcinoma was identified, which was further characterized as high-grade non-muscle-invasive bladder cancer The patient began six cycles of weekly intravesical BCG therapy (TICE^®^ strain). A staging PET-CT scan revealed widespread FDG-avid lesions in the liver, lymph nodes (above and below the diaphragm), and multiple skeletal sites. A liver biopsy confirmed recurrent cHL. Consequently, the intravesical BCG therapy was halted, and after a multidisciplinary team (MDT) discussion, the patient commenced second-line chemotherapy (chlorambucil, vinblastine, procarbazine, and prednisolone [ChlVPP]). A post-treatment PET-CT scan again showed complete metabolic remission.

**Fig. 2:**
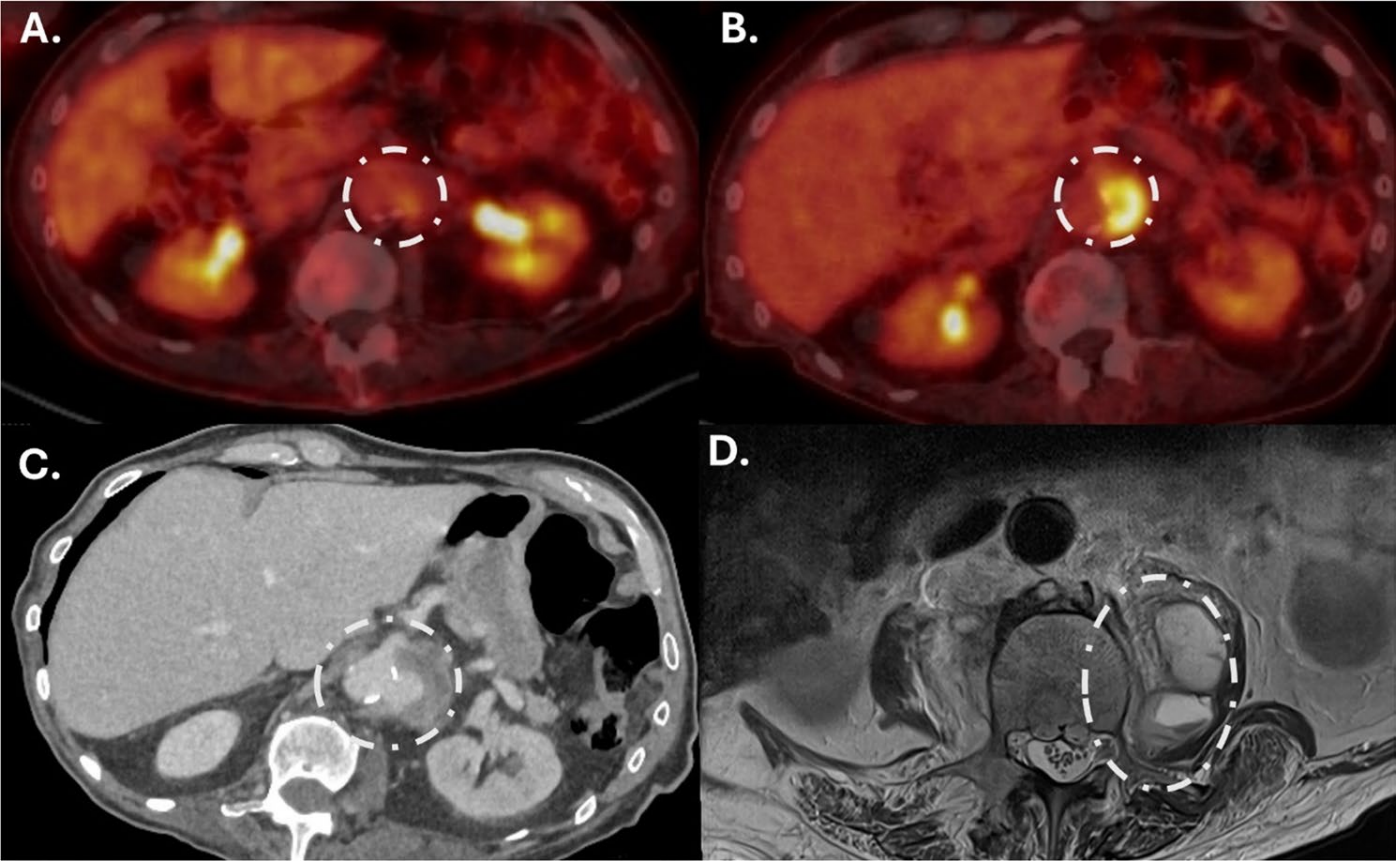
Radiological progression of BCG-associated aortic mycotic aneurysm. **A** Surveillance PET scan in December 2023; **B** surveillance PET scan June 2024 with increased avidity in the left para-aortic region; **C** CT-scan October 2024 with contained aortic aneurysm rupture; **D** MRI scan October 2024 showing left psoas abscess collection

Surveillance cystoscopy in 2023 showed no evidence of invasive bladder cancer. The patient resumed re-induction intravesical BCG therapy, with the same regimen, with maintenance instillations scheduled for 3, 6, 12, 18, 24, 30, and 36 months. A PET scan at the end of 2023 (Fig. 2A) continued to support complete metabolic remission.

In June 2024, the patient experienced significant weight loss (> 10%) and a repeat PET-CT scan (Fig. 2B) identified new retroperitoneal lymphadenopathy with increased avidity (Deauville 5) and an enlarged left para-aortic lymph node. MDT review concluded that this was likely recurrent lymphoma localized to the upper abdomen and due to its location, biopsy was not performed. He began palliative pembrolizumab therapy (200 mg every 3 weeks), which was well tolerated for the first three cycles, except for mild epigastric pain, well-controlled with a proton pump inhibitor. After the third cycle, the patient developed lower back pain, prompting his presentation to the ED.

### Hospital admission and BCG diagnosis

Upon ED presentation for moderate lower back pain, the patient was afebrile, with normal vital signs. His laboratory results included an elevated C-reactive protein (CRP) of 30 mg/L, erythrocyte sedimentation rate (ESR) of 43 mm/hr, and hemoglobin of 107 g/L, with a normal white blood cell (WBC) count (4.1 × 10^9^ cells/L) and mild lymphopenia (0.9 × 10^9^ cells/L). A contrast-enhanced CT scan (Fig. 2C) revealed a 36mm^2^ saccular abdominal aortic aneurysm with an associated left periaortic soft tissue mass, suggestive of an abscess, as well as a low-attenuation foci in the left psoas muscle, and a lytic lesion of the L3 vertebral body. After review in radiology meeting, the differential diagnosis favored a mycotic aneurysm over lymphoma progression. Given concerns about a contained rupture of the aorta, the case was discussed with the vascular surgery team. Due to age, frailty, and prognosis, vascular intervention was deemed inappropriate, and the patient was referred to the palliative and supportive care team for assistance with pain management.

A CT-guided drainage and core biopsy of the left psoas muscle was performed with removal of 8 ml of turbulent fluid. Microscopy showed necrotizing granulomatous inflammation and Ziehl-Neelsen staining demonstrated acid-fast bacilli. Mycobacterium tuberculosis complex (MTB) in-house polymerase chain reaction (PCR) targeting *IS6110* and TB- specific internal transcribed spacer (ITS) region) was positive the following day. Immunohistochemistry did not show Hodgkin lymphoma in the biopsy. The patient had no known risk factors for tuberculosis. Interferon Gamma Release Assay (IGRA) was negative. Mycobacterial blood cultures and urine cultures were sent, without isolation of organisms. Treatment for suspected disseminated BCG infection was initiated with isoniazid (plus pyridoxine), rifampicin, ethambutol (HRE), and moxifloxacin. An echocardiogram was performed, showing mildly reduced left ventricular ejection function and focal hypokinesia, without features of pericarditis. MRI of the lumbar spine (Fig. 2D) confirmed osteomyelitis and discitis in the L2-L4 region with associated inflammatory epidural mass effect, but no abscess. No neurological deficits were observed on clinician examination. Pain management and mobility were optimised, and the patient was discharged home. Anti-mycobacterial therapy continued under the care of the local public health unit.

### Disease progression and outcome

The patient was readmitted 3 weeks later with worsening back pain. On this occasion, he was febrile (38.0 °C), with a further rise in CRP (74 mg/L) but a normal WBC count. A repeat CT scan showed progression of the abdominal aneurysm (42 × 38 mm) with an increase in the size of the left psoas abscess. Empiric antibiotics (gentamicin and vancomycin) were started but discontinued after a negative septic screen. The cultured isolate from the initial psoas aspiration, identified 12 days later, confirmed *Mycobacterium bovis* BCG by Genomic Deletion analysis [7], which was sensitive to HRE. Moxifloxacin was stopped, and repeat CT-guided drainage was performed, with the removal of 10 mL of fluid. A pigtail drain was inserted and drained further 10 ml over 3 days before removal. MTB PCR was positive in this repeat aspirate, and cultures were positive after 25 days.

Following drain insertion, the patient became febrile and delirious and was started on empirical cefepime. Lower limb cellulitis was identified, for which antibiotics were switched to cefazolin for 6 days. Despite high-dose oral opioids, his back pain worsened and became severe. Repeat CT imaging showed further progression of the saccular aneurysm (44 × 40 mm), vertebral osteomyelitis, and re-accumulation of the psoas abscess. After consultation with the palliative care team, the patient was transitioned to subcutaneous opioid analgesia through a syringe driver. He died peacefully in the hospital a few days later.

Immunosuppression screening revealed persistent immunoglobulin A (IgA) hypogammaglobulinemia (0.5 g/L) and low CD4 + count (280 cells/µL) following chemotherapy. Laboratory investigations also showed developing progressive hyponatremia (125mmol/L), with euvolemic fluid status and investigations consistent with the syndrome of inappropriate antidiuretic hormone secretion (SIADH), which improved with fluid restriction.

## Discussion

This case report presents one of the first documented instances of disseminated BCG infection associated with immune checkpoint inhibitor therapy [8], following concurrent treatment for bladder cancer and a hematological malignancy.

Disseminated BCG infection following BCG instillation is uncommon and is estimated to occur at around 1% [2, 9, 10];while in immunosuppressed patients, prevalence may be slightly higher, at approximately 1.8% [11]. The clinical presentation can vary significantly, with disseminated BCG infection manifesting acutely as a sepsis-like syndrome [12], throughout the treatment period, or up to several years after completing therapy [6, 13]; with the mean duration of BCG therapy until diagnosis around 12 months [10]. Whether BCG-related complications are primarily driven by hypersensitivity inflammatory reactions, or active infection, to date remains unclear [14, 15]. An internal review of BCG cases over the last 10 years by the New South Wales (NSW) Mycobacterium Reference Laboratory, which covers a population of approximately eight million people, identified 91 unique BCG isolates. These include several previously reported cases, including two each of mycotic aneurysms and osteomyelitis [16–21]. Five of these cases were identified within our local health district, which has an approximate population of one million [22].

Vascular infections with BCG have been previously documented, as has the association with psoas abscesses and osteomyelitis, likely due to direct spread of BCG infection through tissue planes [23]. In approximately one-third of these cases, patients present with aortic rupture, suggesting a slowly progressive infection that often results in delayed diagnosis [23]. A recent retrospective review in Western Australia found mycotic aneurysm in 2/11 (18.1%) cases of disseminated BCG, with one fatal outcome [24]. In our case, the progression of BCG dissemination was uniquely tracked via PET/CT scans due to surveillance of an underlying hematological malignancy. A retrospective radiological review suggested that disseminated BCG infection was likely established at the time of suspected cHL recurrence (> 2 months prior to onset of back pain), despite minimal symptom burden apart from weight loss and normal inflammatory markers (CRP 4 mg/L) at this time. The patient’s underlying immunosuppression following chemotherapy may have contributed to the development of a favorable environment for BCG dissemination while undergoing maintenance instillations.

The anti-mycobacterial treatment strategy used followed published guidelines, without use of corticosteroids [25]. Additionally, source control with psoas abscess drainage was attempted twice, and showed culture-positivity despite over a month of appropriate treatment. Despite these efforts, the poor clinical outcome in this case supports the likely benefit of surgical or endovascular intervention in patients who are suitable candidates [23].

Immune checkpoint inhibitors have revolutionized cancer treatment, offering therapy options for a wide range of malignancies, including Hodgkin’s lymphoma [26]. The role of immune checkpoint therapy (in this case pembrolizumab, a PD-1 inhibitor) in the progression of established BCG infection is challenging to define. Disseminated BCG infection could have developed independently of immune checkpoint inhibitor therapy, and a causative correlation cannot be established. However, the rapid progression of disease following the initiation of immune checkpoint inhibitor therapy, despite ongoing anti-mycobacterial treatment, is notable. A similar case of disseminated BCG infection has been seen following treatment with pembrolizumab a year after initial intravesical BCG immunotherapy [8]. A phase 1 clinical study examining the combination of BCG (itself an immunotherapy) and immune checkpoint inhibitors in metastatic melanoma reported high-grade immune-related adverse events, suggesting potential enhanced immune activation [15]. Similarly, systemic activation of BCG has been linked to immune recovery following the discontinuation of rituximab treatment [27], as well as immune reconstitution inflammatory syndrome (IRIS) in patients initiating antiretroviral therapy for HIV [28, 29]. The comparative impact of immune checkpoint inhibitors on the development of active tuberculosis remains unclear, though limited studies suggest that the risk may be comparable to or higher than conventional chemotherapy [30], highlighting the need for further longitudinal research [31]. In contrast to these concerns, pembrolizumab is currently undergoing trials for bladder cancer unresponsive to previous BCG instillation [32, 33], as well as in combination with BCG [34–36], suggesting a complex and potentially synergistic interaction between the two therapies when used in the appropriate setting. This is further evidenced by increased tumour PD-L1 expression after BCG exposure [37]. This interplay would require close clinical and radiological monitoring to early determine any adverse effects. To date, only three clinical trials have reported adverse events, with no BCG infection-specific outcomes available (supplementary table S1) [32, 33, 38].

## Conclusion

In conclusion, disseminated BCG infection remains a significant diagnostic challenge that can clinically and radiologically mimic disseminated malignancy. A high clinical suspicion is required by clinicians, especially with a history of known exposure. The risks associated with immune-activating therapies in patients with concealed BCG infections require further investigation.

## Supplementary Information

Supplementary Material 1.

## Data Availability

No datasets were generated or analysed during the current study.

## Abbreviations

BCG: Bacillus Calmette Guérin
cHL: Classical Hodgkin Lymphoma
CRP: C-Reactive Protein
IRIS: Immune Reconstitution Inflammatory Syndrome
PD: Programmed Death
ChlVPP: Chlorambucil, Vinblastine, Procarbazine, Prednisolone
PVAG: Prednisolone, Vinblastine, Doxorubicin, and gemcitabine (PVAG
PET: Positron Emission Tomography
CT: Computer Tomography
PCR: Polymerase chain reaction
MRI: Magnetic Resonance Imaging
HRE: Isoniazid, Rifampicin, Ethambutol
MTB: Mycobacterium tuberculosis Complex
HIV/AIDS: Human Immunodeficiency Virus/Acquired Immunodeficiency Syndrome
MDT: Multidisciplinary team
ED: Emergency Department
WBC: White Blood Cell
ITS: Internal Transcribed Spacer
ESR: Erythrocyte Sedimentation Rate
IGRA: Interferon Gamma Release Assay
SIADH: Syndrome of Inappropriate Antidiuretic Hormone Secretion
NSW: New South Wales
