# Supplementary material for: Disseminated Bacillus Calmette-Guerin infection following concurrent intravesical BCG therapy and immune checkpoint inhibitior therapy: a case report
Source: BMC Infect Dis. 2025 Nov 13;25:1575. doi: 10.1186/s12879-025-11511-3 (PMC12616909; doi:10.1186/s12879-025-11511-3)
Supplement: Supplementary file 1 — Supplementary Material 1. [file 12879_2025_11511_MOESM1_ESM.docx]

Supplementary Table S1. Ongoing and completed clinical trials combining immune checkpoint inhibitors (ICIs) with intravesical BCG therapy for bladder cancer.

| Trial (ClinicalTrials.gov ID) | Therapy | Phase | BCG treatment context | Enrolment* | Status / Years | BCG-related complications |
| --- | --- | --- | --- | --- | --- | --- |
| CREST ^[1, 2]^ (NCT04165317) | Sansalimab (PD-1i) + BCG combinations vs. BCG | 3 | BCG-naïve (Cohort A) | 1055 (1:1:1) | Ongoing (2019–) | Pyrexia (15% in combination group vs. 12% BCG-monotherapy); treatment-related BCG discontinuation due to AE (21.9% vs. 9.7%) |
| KEYNOTE-057 ^[3]^ (NCT02625961) | Pembrolizumab (PD-L1i) | 2 | Following previous BCG therapy | 132 | Ongoing (2016–) | 1 death from systemic infection (aetiology not confirmed as BCG-related) |
| SWOG S1605 ^[4]^ (NCT02844816) | Atezolizumab | 2 | Following BCG-unresponsive NMIBC | 166 | Completed (2017–2019) | No reported BCG-related complications |
| ALBAN ^[5]^ (NCT03799835) | Atezolizumab (PD-L1i) + BCG vs BCG | 3 | BCG-naïve | 517 (1:1) | Ongoing (2019–) | Safety data not yet published |
| POTOMAC (NCT03528694) | Durvalumab (PD-L1i) + BCG combination therapies vs BCG | 3 | BCG-naïve or >3 years since Tx | 1018 (1:1:1) | Ongoing (2018–) | Safety data not yet published |
| KEYNOTE-676 ^[6]^ (NCT03711032) | Pembrolizumab + BCG vs BCG | 3 | Following previous BCG therapy | 1397 | Ongoing (2018–) | Safety data not yet published |
| MSK Cancer Center (NCT03504163) | Pembrolizumab + BCG | 2 | First-line therapy for high-risk NMIBC | 45 | Ongoing (2018–) | Safety data not yet published |
| CheckMate7G8 (NCT04149574) | Nivolumab (PD-1i) + BCG vs. BCG after | 3 | Persistent or recurrent disease, not BCG unresponsive | 12 (terminated early due to low accrual) | Terminated (2019–2023) | Safety data not yet published |

AE: Adverse event; BCG: Bacillus Calmette-Guérin; NMIBC: Non-Muscle Invasive Bladder Cancer; PD-L1i: PD-Ligand 1 inhibitor; PD-1i: PD-1 inhibitor

* Most trials excluded patients with concurrent malignancies, prior BCG-related complications, immunosuppressive conditions, or ongoing immunosuppressive therapy. Published safety data remain limited.
